# Bilateral Below-Knee Amputations in Atherosclerotic Limbs: A Case Report

**DOI:** 10.7759/cureus.71285

**Published:** 2024-10-11

**Authors:** Sawyer S Longley, Cory J Dixon, Aaron P Tillman, Samuel S Maroney

**Affiliations:** 1 Department of Research, Alabama College of Osteopathic Medicine, Dothan, USA; 2 Department of Orthopedics, Abilene Sports Medicine and Orthopedics, Abilene, USA

**Keywords:** amputation revision, below-knee amputation, delayed wound healing, peripheral arterial diseases, pvd: peripheral vascular disease

## Abstract

Peripheral artery disease (PAD) compromises blood flow, often leading to the need for amputation. This case report details a 72-year-old male with a history of PAD and type 2 diabetes mellitus who underwent below-knee amputation (BKA) due to atherosclerotic limb disease. He subsequently faced complications such as wound dehiscence, prolonged wheelchair dependency, and an eventual contralateral BKA. Despite interventions including wound care and revision surgeries, persistent delayed healing underscores the complex interplay between atherosclerosis, diabetes, and wound healing. This patient’s journey highlights the critical importance of tailored management strategies and patient education in mitigating the impact of PAD-related amputations, emphasizing the need for comprehensive care to address the multifaceted challenges posed by vascular diseases.

## Introduction

Peripheral vascular disease (PVD) affects an estimated 8.5 million Americans and over 200 million individuals worldwide [1]. PVD can be categorized as arterial, venous, or mixed, with peripheral artery disease (PAD) specifically referring to reduced arterial blood flow to the extremities, often caused by atherosclerosis [2]. Despite its prevalence and potential for severe clinical outcomes, PAD has frequently been undervalued within the healthcare community, leading to significant gaps in clinical practice, research, and management strategies [3]. The combination of PAD and diabetes presents additional challenges, as affected patients exhibit markedly impaired wound-healing abilities [4]. In fact, individuals with diabetes face a 20-fold increased risk of lower limb amputation compared to the general population [5].

Surgical site infections are a significant concern for vascular patients who undergo major limb amputations, with a higher incidence associated with below-knee amputations (BKAs) than with above-knee amputations [6]. Such infections are more prevalent among diabetic patients, often resulting in prolonged antibiotic use and the need for amputation revision [7].

This case report presents a 72-year-old man who underwent a BKA of his left lower extremity due to atherosclerotic limb disease, complicated by type 2 diabetes and poor self-mobility strategies. The patient experienced wound dehiscence, necessitating revision surgery six months later, which left him wheelchair-bound for an extended period as a unilateral amputee. Over a year after the left BKA revision, gangrene developed on his right foot, resulting in a right BKA. This case underscores the unique challenges faced by patients with vascular diseases during the amputation process.

## Case presentation

A 72-year-old male presented to the clinic with an atherosclerotic left lower extremity exhibiting gangrenous tissue in the foot. Contributing factors to the patient’s clinical presentation included PAD for seven years, type 2 diabetes for 20 years, and hypertension for 15 years. A BKA of the gangrenous left lower limb was performed, and wound care instructions were provided postoperatively.

At the two-week postoperative follow-up, the patient remained non-weight bearing (NWB) in a wheelchair and described his symptoms as dull and aching pain. During this visit, the patient was progressing as expected, with staples removed from the surgical site. A stump shrinker was recommended, and further wound care instructions were provided.

**Figure 1:**
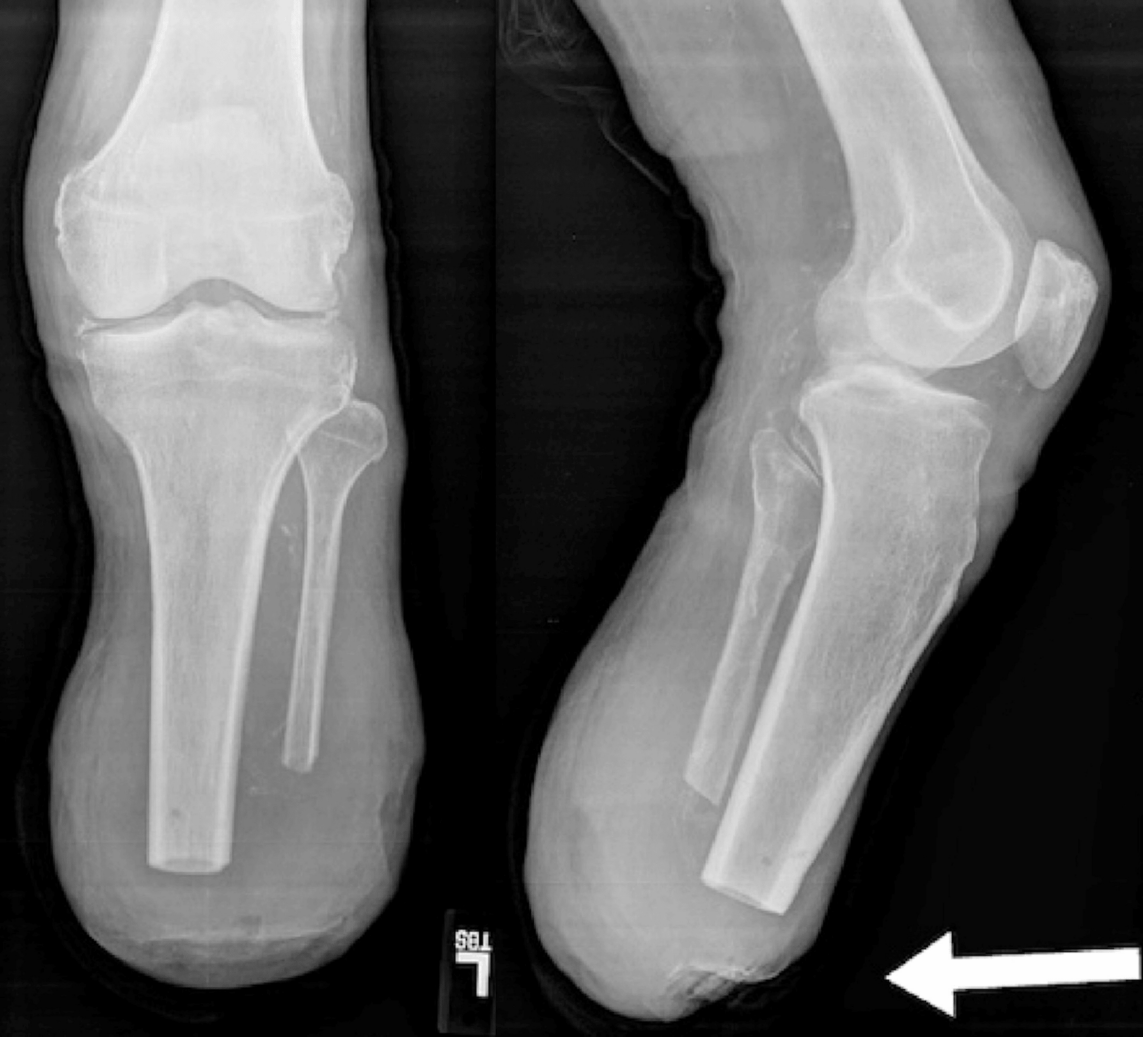
X-rays of the patient’s left tibia and fibula prior to the revision of the BKA X-rays of the patient’s left tibia and fibula revealed an anterior soft tissue defect characterized by skin breakdown, as indicated by the white arrow. BKA, below-knee amputation

At the two-month postoperative follow-up, the patient presented with wound dehiscence after crawling on the floor to navigate his surroundings. He received formal wound care and was reevaluated six weeks later. During this visit, the patient reported worsening and constant pain. An X-ray of his tibia and fibula showed adequate muscle coverage; however, a large soft tissue defect anteriorly at the site of dehiscence was observed, consistent with his physical examination (Figure 1). His wound exhibited significant breakdown, with purulent material noted. Transcutaneous oxygen measurements (TCOM) indicated suboptimal blood flow, and repeat TCOM was recommended, with a follow-up scheduled in three weeks. The patient remained wheelchair-bound and received formal wound care, including a wound vacuum-assisted closure (VAC) device with hyperbaric oxygen therapy. Despite these interventions, the patient was ultimately hospitalized due to wound infection.

While hospitalized, the patient received IV antibiotics, including 1 gram of vancomycin every 12 hours for four weeks and 3.375 grams of piperacillin/tazobactam every eight hours for two weeks. At five months post-BKA, despite the IV antibiotic treatment, adequate distal perfusion noted on follow-up TCOM, and subjective reports of symptom improvement, the patient remained wheelchair-bound with an open wound. Due to incomplete healing, a revision of the left BKA was recommended.

**Figure 2:**
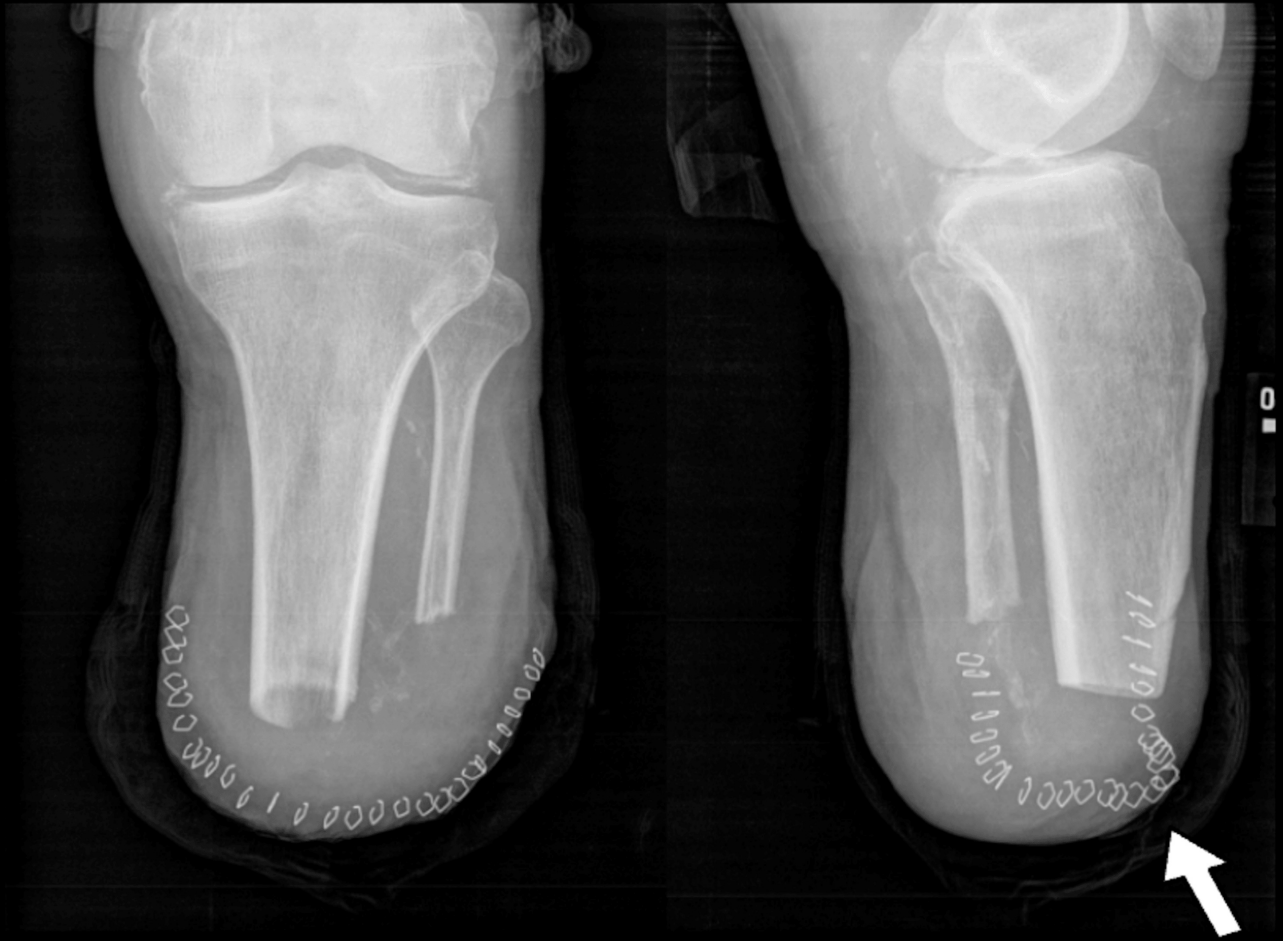
X-rays of the patient’s left tibia and fibula following the revision of the BKA X-rays of the patient’s left tibia and fibula following the BKA revision demonstrated adequate soft tissue coverage of the residual limb anteriorly, as indicated by the white arrow. BKA, below-knee amputation

One month after the revision, the patient’s soft tissue exhibited no signs of breakdown, and he was referred for formal wound management (Figure 2). However, a few months later, the medial side of his wound continued to heal slowly. Home wound care instructions were provided, and the patient was prescribed 100 mg of oral prophylactic doxycycline twice a day for six weeks. He was also instructed to visit a formal wound care center if the wound did not improve within a week.

A month later, the wound showed signs of improved healing, allowing the patient to be fitted for his first prosthetic at the five-month mark following the left BKA revision. One year after his revision surgery, he arrived at the office full weight bearing and ambulating with the assistance of a walker.

A few months later, the patient returned to the office NWB in a wheelchair, presenting a new complaint of right foot pain, swelling, and discomfort. He reported developing gangrene in his right foot several weeks prior. No palpable pulses were noted in the right foot, which appeared very cyanotic. A TCOM study indicated a poor response to the oxygen challenge in the distal right lower extremity. Based on the study results, the decision was made to proceed with a right BKA.

Approximately two months post-right BKA, the patient arrived NWB with the assistance of a wheelchair. As before, he demonstrated delayed healing. Given the events following his prior BKA, an early referral to a wound care specialist was initiated to mitigate the risk of infection. The patient was advised to continue wound care and protect the incision from injury.

At the three-month follow-up after his right BKA, the patient remained NWB with the assistance of a wheelchair. His wound displayed a 4-5 cm area of delayed healing and wound dehiscence along the incision site. Wound management had previously prescribed a wound VAC device prior to the clinic visit. Continued use of the wound VAC was recommended, along with ongoing formal wound care to facilitate improved healing.

**Figure 3:**
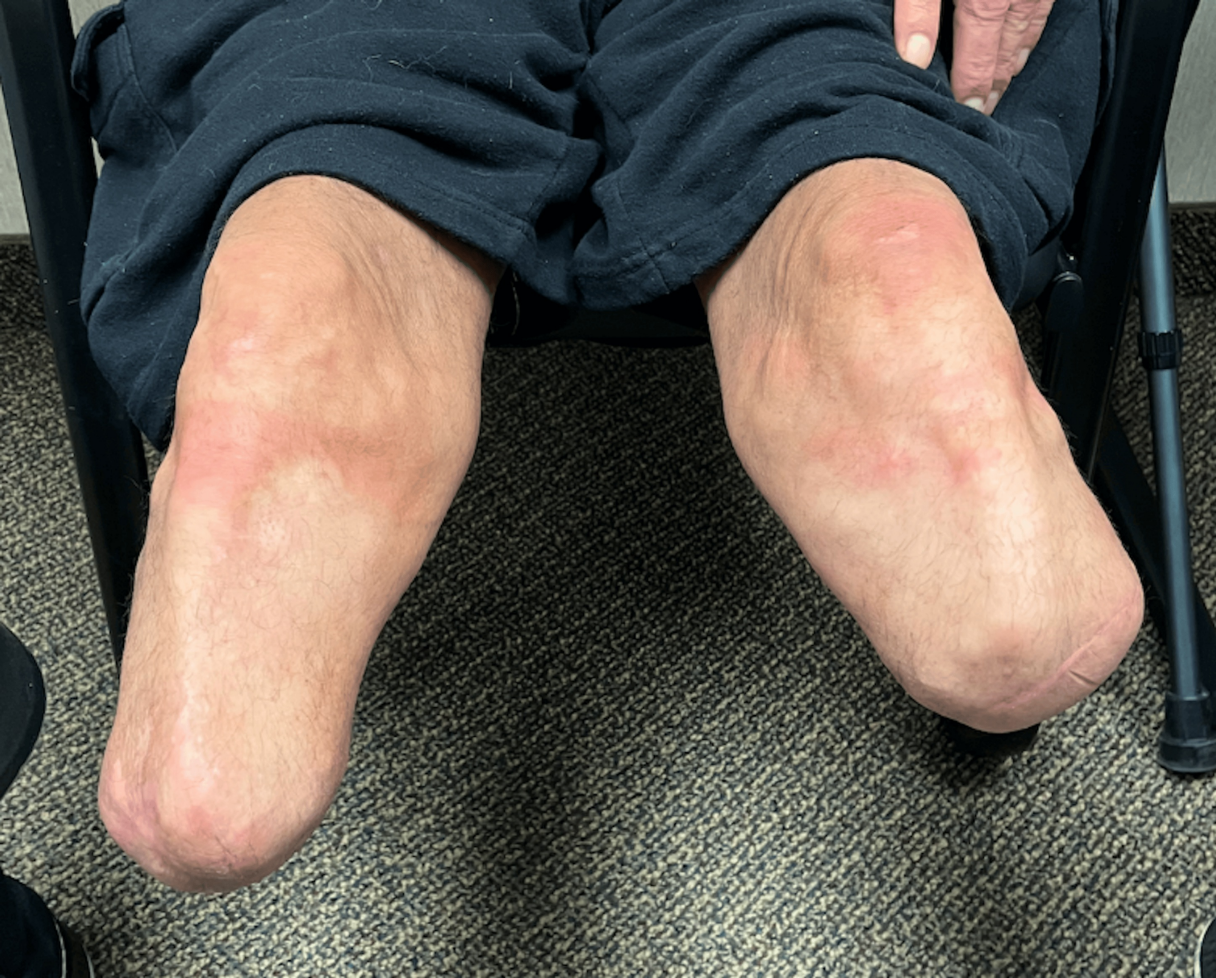
Follow-up visit five years post-bilateral BKA This image illustrates the patient’s bilateral lower limb amputations, demonstrating appropriate healing in both extremities. BKA, below-knee amputation

Over the next few years, with appropriate wound management and follow-up care, the patient’s wounds healed without further issues. At the five-year follow-up from his bilateral BKAs, he was full weight bearing with both wounds healed and bilateral prosthetics in place (Figure 3).

## Discussion

Atherosclerosis is a well-documented systemic disease characterized by plaque formation within vascular beds. This pathological process initiates in the vessel walls, where lipid-laden macrophages contribute to the formation of fatty streaks. Immune cells remain active throughout all stages of plaque development, culminating in the formation of an atheroma surrounded by a smooth muscle cap. Over time, this cap weakens, exposing the thrombotic plaque and leading to platelet aggregation, which can result in thrombus formation. Such a thrombus may occlude the entire vessel, causing ischemia in the affected organ [8].

In the lower limb arteries, atherosclerosis can lead to PAD, particularly in patients with hypertension and diabetes. Those with PAD face an increased risk of major adverse cardiovascular events, including strokes and myocardial infarctions, as well as complications such as delayed wound healing, infection, and foot ulcers. Preventive measures include regular exercise, antiplatelet therapy, lipid-lowering agents, antihypertensive treatment, and antidiabetic medications [3].

Sedentary behavior can exacerbate inflammation, contributing to atherosclerotic buildup in PAD. The most common symptom of PAD - claudication (pain during ambulation that resolves with rest) - restricts mobility and can lead to further sedentary habits [3,9]. This creates a cyclical pattern, exacerbating PAD symptoms. Supervised exercise therapy has shown consistent benefits for patients with PAD, with increased exercise correlating positively with measures of low-flow mediated constriction [10]. For sedentary patients with bilateral BKAs, adherence to medical treatment for hyperlipidemia and diabetes is crucial due to the higher inflammatory burden associated with inactivity, which may exacerbate atherosclerosis-related adverse events. The patient described herein was non-ambulatory for over a year, likely contributing to inflammation and atherosclerotic buildup, along with venous stasis that worsened his PAD in both lower extremities.

Diabetic patients often experience impaired wound healing due to hyperglycemia, which reduces antioxidant enzyme activity and increases reactive oxygen species (ROS) production. This oxidative stress can damage the blood supply and nerve structures [11]. Increased ROS from hyperglycemia further heightens inflammation, endothelial dysfunction, and arterial stiffness, all contributing to lower limb arterial disease development [12]. In this patient, diabetes likely intensified his inflammatory burden, exacerbating atherosclerotic buildup in his lower extremities.

BKA refers to an amputation performed through the tibia and fibula, commonly necessary in patients with PAD. This procedure has a high potential to restore near-normal function, as weight is transferred through the patellar tendon [5]. Research indicates that diabetic patients undergoing BKA typically have a median healing time of about 2.5 months, with 60% becoming ambulatory by approximately 9.5 months [13]. However, the patient in this case report did not achieve proper healing until 10 months post-first BKA and remained non-ambulatory until around 15 months afterward. Unfortunately, BKAs are associated with high mortality rates due to comorbidities such as renal disease, heart disease, dementia, and preoperative non-ambulatory status [14]. This underscores the necessity for patients to adhere to their hyperlipidemia and diabetes medications before and after BKA. Patient education is vital in managing chronic wounds, as effective education and adherence to treatment plans can significantly reduce complications and alleviate healthcare burdens on patients [15]. In this instance, the patient dragged his wound on the floor immediately post-BKA, likely contributing to wound dehiscence, infection, delayed healing, and the need for subsequent revision surgery.

## Conclusions

Atherosclerosis is a prevalent underlying condition that can lead to PAD. The combination of atherosclerosis and diabetes can significantly exacerbate inflammation and endothelial dysfunction within vessel walls. In this case, we present a 72-year-old man who underwent a left BKA due to PAD, which was complicated by impaired wound healing, wound dehiscence, and diabetes. His condition resulted in being wheelchair-bound for over a year, likely contributing to the development of venous stasis in his right lower extremity. This venous stasis likely led to gangrene in the right lower extremity, necessitating a subsequent BKA of that limb. This case underscores the unique challenges faced by patients with diabetes and atherosclerosis due to the complex interplay between these diseases.
